# Comparison of Colorectal and Gastric Cancer: Survival and Prognostic Factors

**DOI:** 10.4103/1319-3767.43284

**Published:** 2009-01

**Authors:** Bijan Moghimi-Dehkordi, Azadeh Safaee, Mohammad R. Zali

**Affiliations:** Research Center of Gastroenterology and Liver Diseases, Shahid Beheshti University of Medical Sciences, Tehran, Taleghani Hospital, Tabnak St., Yaman Ave., Velenjak, Tehran, Iran

**Keywords:** Colorectal cancer, gastric cancer, Iran, prognosis, survival rates

## Abstract

**Background/Aims:**

Gastric and colorectal cancers are the most common gastrointestinal malignancies in Iran. We aim to compare the survival rates and prognostic factors between these two cancers.

**Methods:**

We studied 1873 patients with either gastric or colorectal cancer who were registered in one referral cancer registry center in Tehran, Iran. All patients were followed from their time of diagnosis until December 2006 (as failure time). Survival curves were calculated according to the Kaplan-Meier Method and compared by the Log-rank test. Multivariate analysis of prognostic factors was carried out using the Cox proportional hazard model.

**Results:**

Of 1873 patients, there were 746 with gastric cancer and 1138 with colorectal cancer. According to the Kaplan-Meier method 1, 3, 5, and 7-year survival rates were 71.2, 37.8, 25.3, and 19.5%, respectively, in gastric cancer patients and 91.1, 73.1, 61, and 54.9%, respectively, in patients with colorectal cancer. Also, univariate analysis showed that age at diagnosis, sex, grade of tumor, and distant metastasis were of prognostic significance in both cancers (*P* < 0.0001). However, in multivariate analysis, only distant metastasis in colorectal cancer and age at diagnosis, grade of tumor, and distant metastasis in colorectal cancer were identified as independent prognostic factors influencing survival.

**Conclusions:**

According to our findings, survival is significantly related to histological differentiation of tumor and distant metastasis in colorectal cancer patients and only to distant metastasis in gastric cancer patients.

Malignancy is a major health problem in many countries throughout the world.[1] Nowadays, gastrointestinal-related cancers especially gastric cancer (GC) and colorectal cancer (CRC), form a vast bulk of overall malignant conditions. GC is one of the major causes of cancer-related death in the world, even though its incidence has decreased over the past decade.[2] The prognosis of GC is generally poor, especially in Western countries,[3,4] where the overall survival rate at 5 years has not changed, oscillating between 8 and 26%, even though the resectability rate has increased (currently 60–80%).[5–9]

In recent years, cancer morbidity and mortality has increased in Iran, with GC becoming the second most common among all cancers.[10,11] Similarly, CRC is the fourth commonest form of cancer occurring worldwide, with an estimated 783,000 new cases diagnosed in 1990, the most recent year for which international estimates are available.[12] CRC is the third most common cause of cancer-related death in the world.[13] The incidence of CRC in Iran is lower than that in Western countries, being the fifth and third most common cancer in men and women, respectively. However, its incidence in Iran is rising and has therefore become a significant public health issue.[14] Several variables representing pathological, clinical, and therapeutic characteristics have already been studied in numerous retrospective reports in an attempt to identify prognostic indicators in patients with GC[15–21] and CRC.[22–34] The aim of the present study is to compare the survival rates and prognostic factors in GC and CRC in one referral cancer registry in Iran.

## PATIENTS AND METHODS

Between December 21, 2001 and December 21, 2006, 1873 patients with GC or CRC were registered in the cancer registry center of Research Center of Gastroenterology and Liver Disease (RCGLD), Shahid Beheshti Medical University; Tehran, Iran. Data on both cancers were collected using cancer registry forms and medical records of the patients. Two types of variables were analyzed in the survival analysis: (1) demographic variables including sex, age, family history of cancer and (2) clinical variables including histologic grade (degree of tumor differentiation) and presence of metastasis.

All patients were followed from their diagnosis until December 21, 2007 (as failure time) by telephone, and survival times were calculated in months. The survival curves were calculated according to the Kaplan-Meier Method and compared by the Log-rank test. Multivariate analysis of prognostic factors was carried out using the Cox proportional hazard model. SPSS.V.13 was used for all statistical calculations, and *P* < 0.05 was considered as statistically significant.

## RESULTS

Of the 1873 patients identified, 746 (39.8%) were with GC and 1127 (60.2%) were with CRC; 1220 were male (71% GC vs 61.2% CRC) and 653 were female (29.0% GC vs 38.8% CRC). Survival information was not available in 11 patients, and they were excluded from the study. The mean patient age at diagnosis was 55.9 ± 14.8 (range 14 - 94) years. The mean age of patients with GC was 59.63 ± 12.89 years compared with 53.59 ± 14.35 years in patients with CRC (*P* < 0.0001).

The mean age of male patients with GC (60.50 ± 12.55 years) and CRC (54.33 ± 14.57 years) was significantly different (*P* < 0.001). Similarly, the mean age of female patients with GC (57.45 ± 15.50 years) and CRC (52.42 ± 13.91 years) was significantly different (*P* < 0.001).

In GC patients, most (216 or 46.2%) were diagnosed with poorly differentiated grade of tumor, but in CRC patients, most tumors were well-differentiated (443 or 55.5%). Also, 179 (25.5%) of GC and 394 (36.7%) of CRC cases reported a positive family history of cancer. Distant metastasis was seen in 184 (36.4%) of GC and 171 (22.3%) of CRC patients [Table 1]. Mean of survival time of patients were 42.46 (CI, 35.74–49.17) for GC and 104.99 (CI, 94.96–115.02) for CRC cases, respectively.

**Table 1:** Clinical characteristics of patients with GC and CRC

Variables		GC* patients		CRC† patients	
		n	%	n	%
Age at diagnosis	≤50	182	24.5	482	42.8
	>50	560	75.5	645	57.2
Sex	Male	530	71.0	690	61.2
	Female	216	29.0	437	38.8
Family history of cancer	Have	179	25.5	394	36.7
	Not have	523	74.5	679	63.3
Grade of tumor	Well diff.	112	23.9	443	55.5
	Moderately diff.	140	29.9	285	35.7
	Poorly diff.	216	46.2	70	8.8
Pathologic distant metastasis	Have	184	36.4	171	22.3
	Not have	322	63.6	595	77.7

* Gastric cancer

† Colorectal cancer

**Figure 1:**
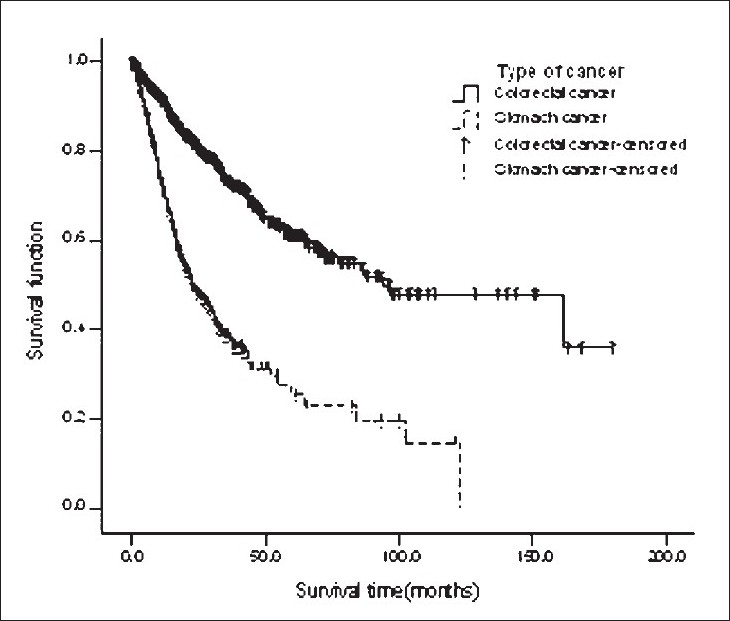
Survival curve of GC and CRC patients using Kaplan-Meier method.

Survival of patients with GC was less than those with CRC (*P* < 0.0001) [Figure 1]. Univariate analysis showed that the factors influencing overall survival rate in both GC and CRC patients were age at diagnosis, sex, grade of tumor, and pathologic distant metastasis (*P* < 0.0001). In relation to the age at diagnosis, CRC patients have better survival than GC patients in all age groups (*P* < 0.001). Also, male patients with CRC have a longer life when compared with those with GC (*P* < 0.001).

Univariate analysis showed that CRC patients have higher survival rates than GC patients, regardless of the degree of tumor differentiation (well-differentiated, moderately-differentiated, poorly differentiated). Also, a better survival was seen in CRC cases with distant metastasis when compared with GC cases of the same status.

**Figure 2:**
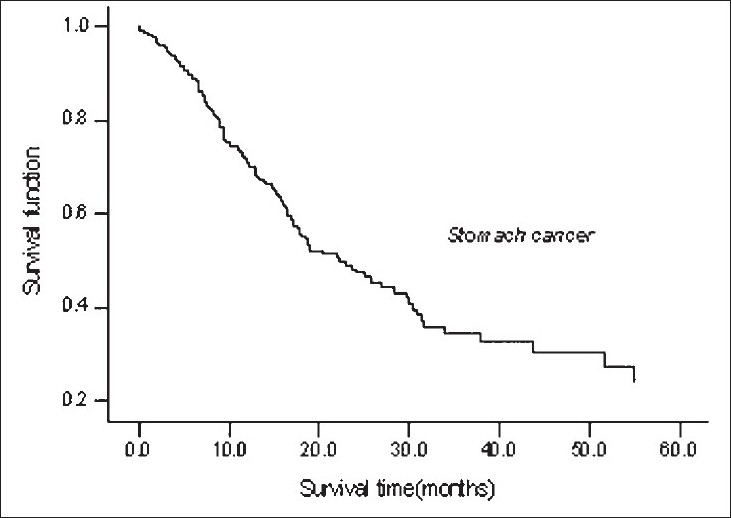
Survival curve of GC patients using Cox proportional hazard model.

**Figure 3:**
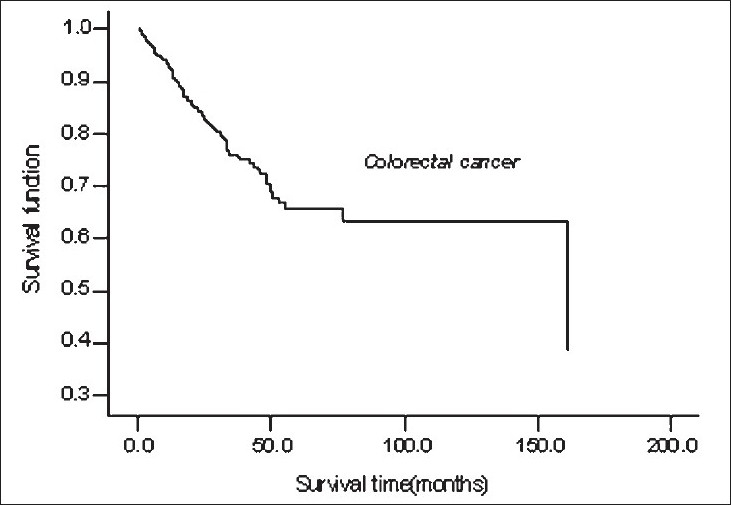
Survival curve of CRC patients using Cox proportional hazard model.

Multivariate analysis showed that grading of tumor and distant metastasis of tumor in CRC patients, and only distant metastasis in GC patients, were the most important prognostic factors determining survival. Poorly and moderately-differentiated tumors exhibited an increased risk of death of 2.18 and 1.71-fold than well-differentiated tumors in CRC patients. Likewise, patients with distant metastasis in GC and CRC have hazard rates of 2.25 and 1.92, respectively [Table 2 and Figures 2 and 3].

**Table 2:** Prognostic factors in GC and CRC patients using Cox proportional hazard model

Type of cancer	Variable	Subgroups	Hazard rate	CI (95%)	
				
				Lower	Upper
Colorectal cancer	Pathologic distant metastasis	Not have*	1	–	–
		Have	1.923	1.263	2.928
	Grade of tumor	Well differentiated*	1	–	–
		Moderately differentiated	1.711	1.164	2.515
		Poorly differentiated	2.185	1.228	3.890
Gastric cancer	Pathologic distant metastasis	Not have*	1	–	–
		Have	2.251	1.555	3.259

* Reference group

## DISCUSSION

GC and CRC are two of the most commonly prevalent malignancies in Iran. If these cancers were diagnosed at an early stage, patients could have a highly favorable prognosis and avoid extended surgery, which may produce complications, especially in the elderly people.[35]

The data used in this study were collected in a cancer registry belonging to the Research Center of Gastroenterology and Liver Diseases of Shahid Beheshti University of Medical Sciences, Tehran, Iran. This cancer registry serves as a major referral registry for the region.

Prognostic factors in GC[36–41] and CRC[42–51] are a source of controversy in many series. This study, based upon a prospective database, indicates that several patient- and tumor-related factors affect survival but are not independent prognostic factors. Compared with CRC, GC has a dismal prognosis and a low 5-year survival rate.

It is generally believed that young patients with CRC have a worse survival rate. Reports from Europe demonstrate that the 5-year survival rate for patients (30 years old or younger) is only 25-30%.[52,53] Young patients are more likely to present with late-stage disease. The young patients also have higher-grade tumors[54] About 60-67% of young patients with CRC have a later stage (III/IV) disease,[53,55] most of which are poorly differentiated or mucinous tumors[53–57] indicating a very poor prognosis. Other reports demonstrate that the 5-year survival rate of patients 30 years old or younger is around 40%.[58]

Also, some authors[59–62] consider that age has prognostic value because young patients with GC have a poorer prognosis than older patients. Perhaps they present with more advanced disease because the index of suspicion for malignant disease is low and so the symptoms are allowed to progress for a longer period before investigation is considered, or there may be a greater biological activity of the tumor, which is more likely to be of the Laur’en diffuse type.

In the present study, in the evaluation of demographic features, age at diagnosis was strongly associated with prognosis in the univariate analysis for both CRC and GC, but was not a significant prognostic factor in multivariate method for both cancers. In all age groups (≤ or > 50 yrs), CRC patients have a longer life than GC patients. Our results are in agreement with most other reports on GC[63–66] and CRC[67,68] which suggest that age at diagnosis significantly affects patient outcome.

Survival analysis indicated that gender is a prognostic factor for GC and CRC patients in the present study. Others have reported that, female patients with CRC compared with males showed a better outcome in the univariate analysis, but the overall survival was not significantly affected by these factors in the multivariate analysis. Our findings are in agreement with most series,[69–72] though some controversies exist.[24,73]

A study conducted in USA stated that GC females had better survival rates and that this was more apparent for early-stage tumors.[74] Curtis *et al*[75] reported that prognosis was better in females in their study, depending on the age and stage of diagnosis. However, no difference with respect to gender was observed in the survival rates for early-stage patients. Male patients had higher mortality rates in a study carried out on patients with GC in Canada[76] when the results were assessed regarding the gender for patients at the same stage (early stage). Another study reported no differences in survival rates regarding gender in patients at early or advanced stages. A study on 2773 patients with GC by the Rotterdam Cancer Registry reported similar resection rates for male and female patients. However, the same study demonstrated significantly lower postoperative mortality rates in female patients.[77] Jin-Pok Kim *et al*[78] stated that female patients had better prognosis in their study carried out on GC patients. Also a study stated no statistical differences in survival of patient on the basis of gender.[79]

With respect to the degree of cellular differentiation, the best prognosis has been found in well-differentiated groups in both tumors.[8,9,21,25,59] Patients with low-grade tumors had a greater survival rate than those with high-grade tumors, the difference being statistically significant.

Another important factor of prognosis was this study is distant metastasis. In univariate and multivariate analysis of both cancers, as expected, patients with metastasis to other organs have a poorer outcome. On the other hand, patients with distant metastasis had a risk of death about 2.25 and 1.92-fold of those without metastasis in GC and CRC, respectively. There are many reports that confirm our finding on GC[80–82] and CRC.[42,44,49–51,83]

This study has some limitations as imposed by the retrospective nature of registry obtained data. For instance, we did not have access to important patient data such as macroscopic tumor type, depth of tumor invasion, and frequency of lymph node involvement. Also, no information was available on the rates of curative resection for the reason that patients under study were operated upon in different hospitals.

Although this study has highlighted the pertinent epidemiological and clinicopathological features of GC and CRC in Iran, further studies are needed to evaluate the environmental risk factors, incidence, the treatment outcomes, and long-term survival outcomes.

## CONCLUSION

According to our findings, survival rates in GC patients were lower than those in CRC patients. High prevalence of GC in Iran and its poor prognosis suggests that health care policy makers should be privy to such data for implementation of screening programs and resource allocation. Also, prognosis of disease is significantly related to histological differentiation of tumor and distant metastasis in CRC patients and only to distant metastasis in GC patients. It appears that these factors are associated with late diagnosis of disease, and therefore, planning and enforcement of screening programs is necessary for an early detection of these cancers.
